# Four Categories of Viral Infection Describe the Health Status of Honey Bee Colonies

**DOI:** 10.1371/journal.pone.0140272

**Published:** 2015-10-08

**Authors:** Esmaeil Amiri, Marina Meixner, Steen Lykke Nielsen, Per Kryger

**Affiliations:** 1 Department of Agroecology, Aarhus University, Slagelse, Denmark; 2 LLH, Bieneninstitut Kirchhain, Kirchhain, Germany; University of Cologne, GERMANY

## Abstract

Honey bee virus prevalence data are an essential prerequisite for managing epidemic events in a population. A survey study was carried out for seven viruses in colonies representing a healthy Danish honey bee population. In addition, colonies from apiaries with high level *Varroa* infestation or high level of winter mortality were also surveyed. Results from RT-qPCR showed a considerable difference of virus levels between healthy and sick colonies. In the group of healthy colonies, no virus was detected in 36% of cases, while at least one virus was found in each of the sick colonies. Virus titers varied among the samples, and multiple virus infections were common in both groups with a high prevalence of Sacbrood virus (SBV), Black queen cell virus (BQCV) and Deformed wing virus (DWV). Based on the distribution of virus titers, we established four categories of infection: samples free of virus (C = 0), samples with low virus titer (estimated number of virus copies 0 < C < 10^3^), samples with medium virus titer (10^3^ ≤ C < 10^7^) and samples with high virus titer (C ≥ 10^7^). This allowed us to statistically compare virus levels in healthy and sick colonies. Using categories to communicate virus diagnosis results to beekeepers may help them to reach an informed decision on management strategies to prevent further spread of viruses among colonies.

## Introduction

Honey bee colonies provide valuable pollinators that enable secure crop productivity [1]. The decline of pollinators in many countries has received considerable public and scientific attention in the past decade [2]. In particular, honey bees have been experiencing considerable colony losses worldwide [3], with yearly estimates of about 30% in the U.S. since 2006 [4–6]. Losses have also been reported from Europe, although Europe appears to experience generally lower levels of losses at a less constant rate [7–9]. Multiple agents, environmental and biological, have been highlighted in correlation to colony losses [3, 7, 10–12]. However, there exists as yet no comprehensive conclusion about the cause or the most probable combination of causes [6, 13, 14].

The ectoparasitic mite, *Varroa destructor*, originating from one of the Asian honey bee species (*Apis cerana*) is an invasive species on the European honey bee (*Apis mellifera*) [15] and considered one major cause responsible for colony losses [4, 11, 12, 16]. The mites puncture the cuticle of honey bee workers and pupae to suck hemolymph, which may lead to immunosuppression of the parasitized host [11, 17, 18], and can also transfer virus particles [11, 19]. The detrimental impact of an infestation with high numbers of *Varroa* mites together with closely associated viruses (DWV, Acute bee paralysis virus (ABPV), Kashmir bee virus (KBV), and Israeli acute paralysis virus (IAPV)) on individual bees as well as colony survival has been established in previous studies [17, 19–22]. In addition to *Varroa* mites, the microsporidian *Nosema ceranae*, another possible agent involved in collapse of honey bee colonies, also created concern in some countries [12, 23, 24]. Infections with the related species *Nosema apis* have been linked with infections of BQCV [25].

Numerous viruses of honey bees are known and occur in different geographical regions [26, 27]. Most surveys focus on DWV, and the ABPV complex (including KBV and IAPV [28]), since they are closely associated with and transmitted by the *Varroa* mite. Furthermore, three viruses for which *Varroa* seems to play no significant role in transmission, namely Chronic bee paralysis virus (CBPV), SBV, and BQCV are frequently surveyed, too [29–31].

Based on results of infection studies, these seven viruses can be characterized as either acute or persistent. In particular, the three closely related viruses from the family Dicistroviridae (ABPV, KBV and IAPV) and the yet unclassified virus (CBPV) can cause acute infection of adult bees with a high rate of viral replication leading to high mortality of workers within a short time span [28, 32]. These viruses are found infrequently; in most prevalence studies they have been detected at low titers from bees without obvious symptoms that were sampled from healthy looking colonies. In contrast, DWV and SBV from the family Iflaviridae and BQCV from the Dicistroviridae family are known to be near omnipresent and persistent viruses, often characterized by an absence of clear disease symptoms [26]. However, they occasionally turn problematic in association with specific biotic and abiotic stress factors [13, 25, 33, 34]. Results from honey bee virus studies reveal that only a minor fraction of the honey bee populations are free of persistent viruses throughout the year [29–31]. These observations support the theory that coevolution between viruses and their hosts may lead towards less malign virus strains [35, 36]. This allows both virus and host to sustain a host-parasite relationship, for mutual persistence. It has been shown for IAPV that some colonies apparently are able to cope with the virus [37, 38]. In consequence, selection and breeding from colonies resistant to pests and pathogens can lead to a sustainable solution to combat honey bee disease [39, 40], since any chemical treatment comes with the risk of inducing resistance in the pathogens and a possible contamination of hive products.

For example, efforts to breed honey bee strains that are more tolerant towards *Varroa* mites are being conducted in several countries [41–43]. In Denmark, a selection program to reduce the impact of Nosemosis has been carried out over two decades which resulted in a strain of Nosema-tolerant bees [44, 45]. The ability to reliably quantify the mites and *Nosema* spores enables beekeepers and scientists to monitor the prevalence and quantity of parasites easily and select the best colonies for the next generations.

For virus diagnosis, quantitative RT-PCR is an advanced and more sensitive technology than qualitative gel-based technique [46]. The ability of RT-qPCR to generate accurate quantitative data has had a positive impact on honey bee viral diagnosis and on our understanding of the problems associated with viral infection [30, 47, 48]. In contrast to the qualitative techniques, quantification allows us to categorize the findings according to the level of viral load.

Colony losses in Denmark reached up to 32% in the winter of 2007 to 2008 and were largely attributed to honey bee viruses, *Varroa* mites and interactions between *Varroa* and viruses [13, 31, 49]. It was found that both high viral titers and the proportion of sick workers within colonies are directly correlated with the number of *Varroa* mites [13]. However, the diagnostic data of this study were obtained from symptomatic colonies and are thus likely to over-represent the prevalence of viral infections in the population. The present study aims to survey seven viruses in the healthy Danish honey bee population during the spring of 2012, to generate baseline data of virus levels in healthy and sick colonies after a long winter. The application of highly sensitive quantitative RT-PCR enables detection of virus titers much below those observed in earlier studies based on qualitative techniques and, thus, contributes to improving prevalence data [31, 49]. In addition, quantitative results may easily be categorized and thus become accessible to statistical evaluation. For beekeepers and breeders, categorized results are more easily understandable and consequently can be translated into management and selection decisions.

## Material and Methods

### Honey bee samples

**Fig 1:**
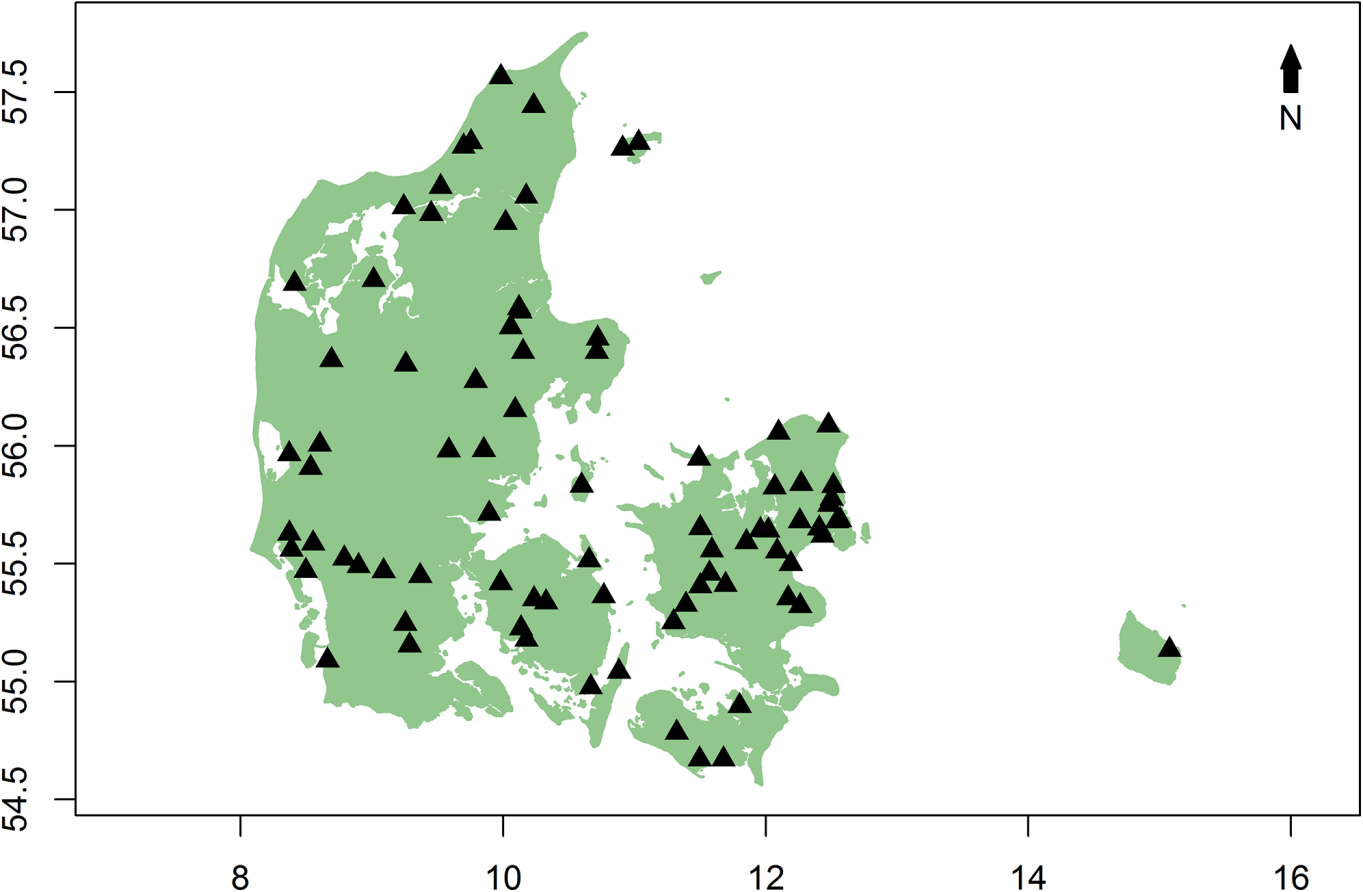
Geographic distribution of honey bee samples in Denmark.

The survey was designed to determine viral presence from apparently healthy honey bee colonies all over Denmark (Fig 1). In total, 241 samples (further referred to as healthy colonies) from 98 apiaries were received. The samples were sent by skilled Danish beekeepers, bee health inspectors, and breeders of queens, all trained in field diagnosis. Each beekeeper was asked to send a standard queen cage with up to 20 live bees per colony, exclusively from colonies that had low levels of *Varroa* mites and were determined free of symptomatic diseases (European Foulbrood, American Foulbrood and Chalkbrood) based on thorough visual inspection. We pooled each sample of 20 bees, which is considered sufficient for a reliable quantification of virus levels in a colony [50]. For comparison, 28 additional samples (further referred to as sick colonies) were collected from the colonies at Flakkebjerg (n = 12) (Department of Agroecology, Aarhus University) that suffered from a heavy infestation with mites and from an apiary (n = 16) with high colony mortality in the previous winter. All samples were collected alive and sent via mail during the spring of 2012 (from mid-April to mid-June). Upon arrival bees were frozen and stored at -80°C until RNA extraction.

### Molecular approaches

Bees of each sample were placed in a 15 mL plastic bottle together with 5–10 steel bearing balls. Using a technique adapted from plant virology, the samples were freeze dried, homogenized in a genogrinder and thereafter, RNA was extracted according to the manufacturer’s manual (for details see [13]). Following RNA extraction, a two-step real-time RT-PCR assay was used to detect and quantify seven honey bee viruses, BQCV, CBPV, SBV, DWV, ABPV, KBV, and IAPV. The three closely related viruses of the ABPV complex (ABPV, KBV and IAPV) were detected in a single assay (AKI) [51]. The housekeeping gene, β-Actin, was used as an internal control, where the presence and quantification of this reference gene ensured that the entire procedure from extraction to quantification was done without degradation of RNA [13].

Quantitative PCR amplifications were carried out on a vii7 apparatus (Applied Biosystems) in duplicate for each sample using SYBR Green DNA binding dye. Final volumes of 12μL with a primer concentration of 0.4μM were loaded on optical 384 well PCR plates. Primers [30, 47, 51–53] used in this study are listed in Table 1.

**Table 1:** Primers used to establish the standard curve and qRT-PCR

Source	Primers name	Primer sequence	Product size(bp)	Reference
**AKI**	F-AKI	5’-CTTTCATGATGTGGAAACTCC	100bp	[51]
	R-AKI	5’-AAACTGAATAATACTGTGCGTA		
**DWV**	F-DWV	5’-GGATGTTATCTCCTGCGTGGAA	69bp	[30]
	R-DWV	5’-CTTCATTAACTGTGTCGTTGATAATTG		
**BQCV**	BQCV-qF	5’-AGTGGCGGAGATGTATGC	294bp	[52]
	BQCV-qB	5’-GGAGGTGAAGTGGCTATATC		
**SBV**	F-SBV	5’- ACCAACCGATTCCTCAGTAG	258bp	[53]
	R-SBV	5’- TCTTCGTCCACTCTCATCAC		
**CBPV**	F-CBPV	5’-CGCAAGTACGCCTTGATAAAGAAC	101bp	[47]
	R-CBPV	5’-ACTACTAGAAACTCGTCGCTTCG		
**β.Actin**	F-β-Actin	5’-TGCCAACACTGTCCTTTCTGGAGGT	96bp	[13]
	R-β-Actin	5’- TTCATGGTGGATGGTGCTAGGGCAG		

### Calibration curve and data analysis

For each virus genome, a standard curve was calculated by plotting the serial dilutions of known amounts of the amplification product against the corresponding Ct values as described previously [13]. Slope and intercept of each curve were calculated with a correlation coefficient of 0.99. The amplification curve for β-Actin was used to confirm that the integrity of the RNA was preserved during the entire procedure, from the preparation of samples and RNA extraction to RT-qPCR (data not shown). Virus loads in each sample were quantified using the absolute quantification method described before [13]. Fisher’s exact test was applied to estimate the variation between healthy and sick colonies. Analysis of the data and visualization were performed using the softwares; Highcharts and R [54].

## Results

### Observed frequencies of the viruses in the population

**Fig 2:**
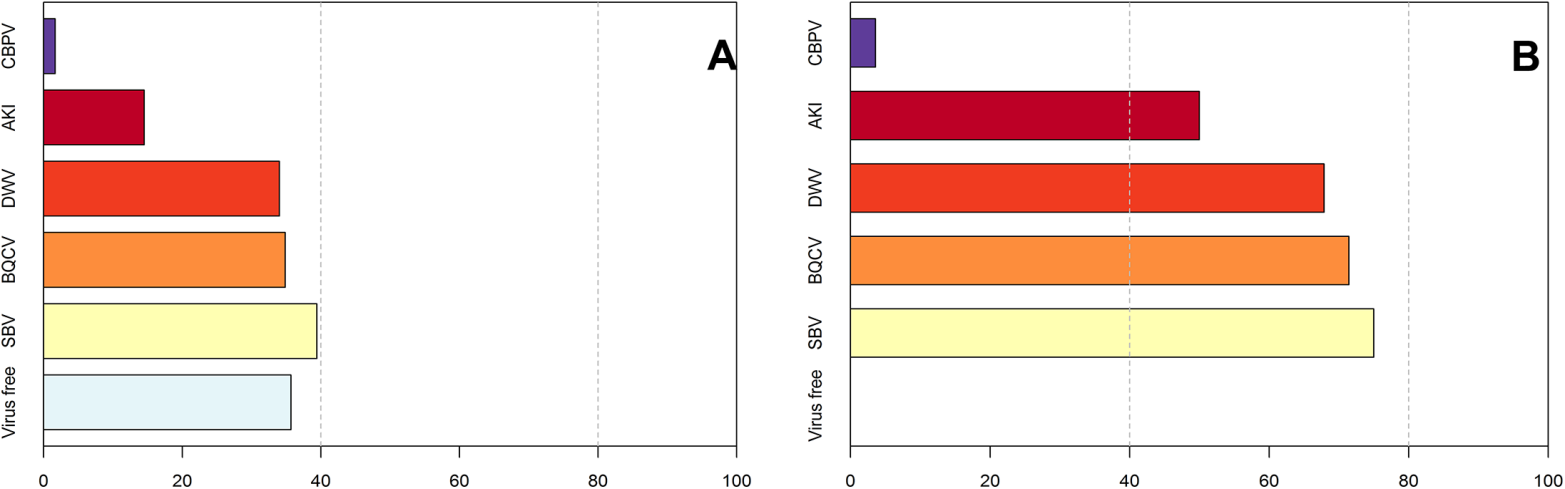
Percentage of different viruses in healthy colonies (A) and sick colonies (B).

A total of 241 apparently healthy and 28 sick colonies were screened for SBV, BQCV, DWV, CBPV and ABPV complex viruses. No virus at all was detected in 36% (n = 86) of the healthy colonies, while all of the sick colonies had at least one virus. The frequencies of the viruses in the two groups of colonies are shown in Fig 2.

The most prevalent viruses in the healthy colonies are SBV, BQCV and DWV, with frequencies of 39% (n = 95), 35% (n = 84) and 34% (n = 82), respectively. These three viruses are also highly prevalent and frequent (75%, 71% and 68%) in the sick colonies. In contrast, viruses of the ABPV complex are present in only 14% of the healthy colonies, but 50% of the sick colonies. CBPV was only observed in four healthy colonies and one sick colony.

**Fig 3:**
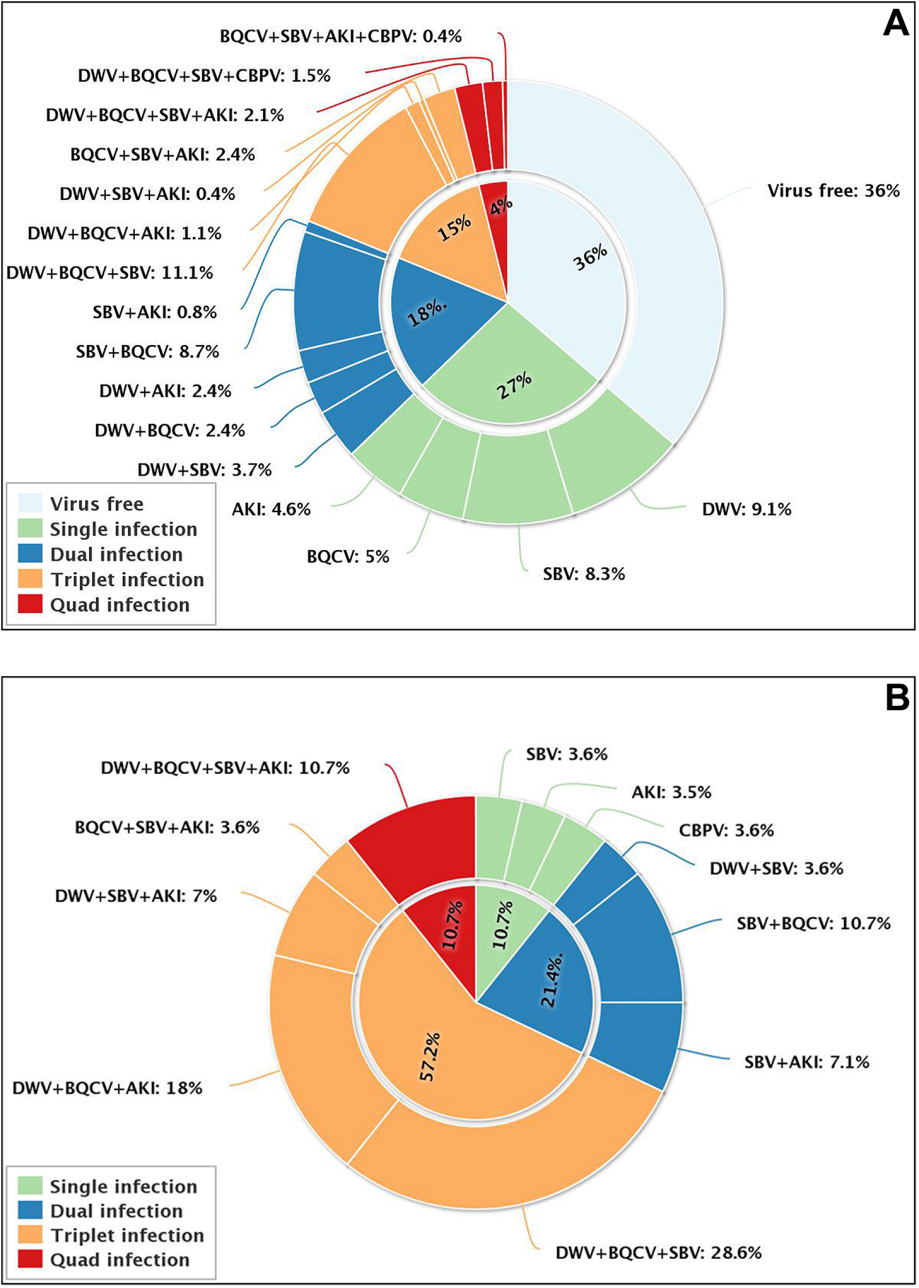
Percentage of single and multiple infections in healthy colonies (A) and sick colonies (B).

Twenty-seven percent of the healthy colonies contained at least one virus, mainly SBV and DWV. Multiple virus infections are also not uncommon in healthy colonies. We observed high rates of duplicate and triplicate infections (18% and 15%, respectively), the majority of them with DWV, SBV and BQCV (Fig 3). As expected, multiple virus infections are very common in sick colonies, with more than half of the samples (57.2%) simultaneously infected by three viruses. Compared to the healthy colonies, the rate of double and quadruple infections in the sick colonies was also much higher (Fig 3).

### Viral titers in the populations

**Fig 4:**
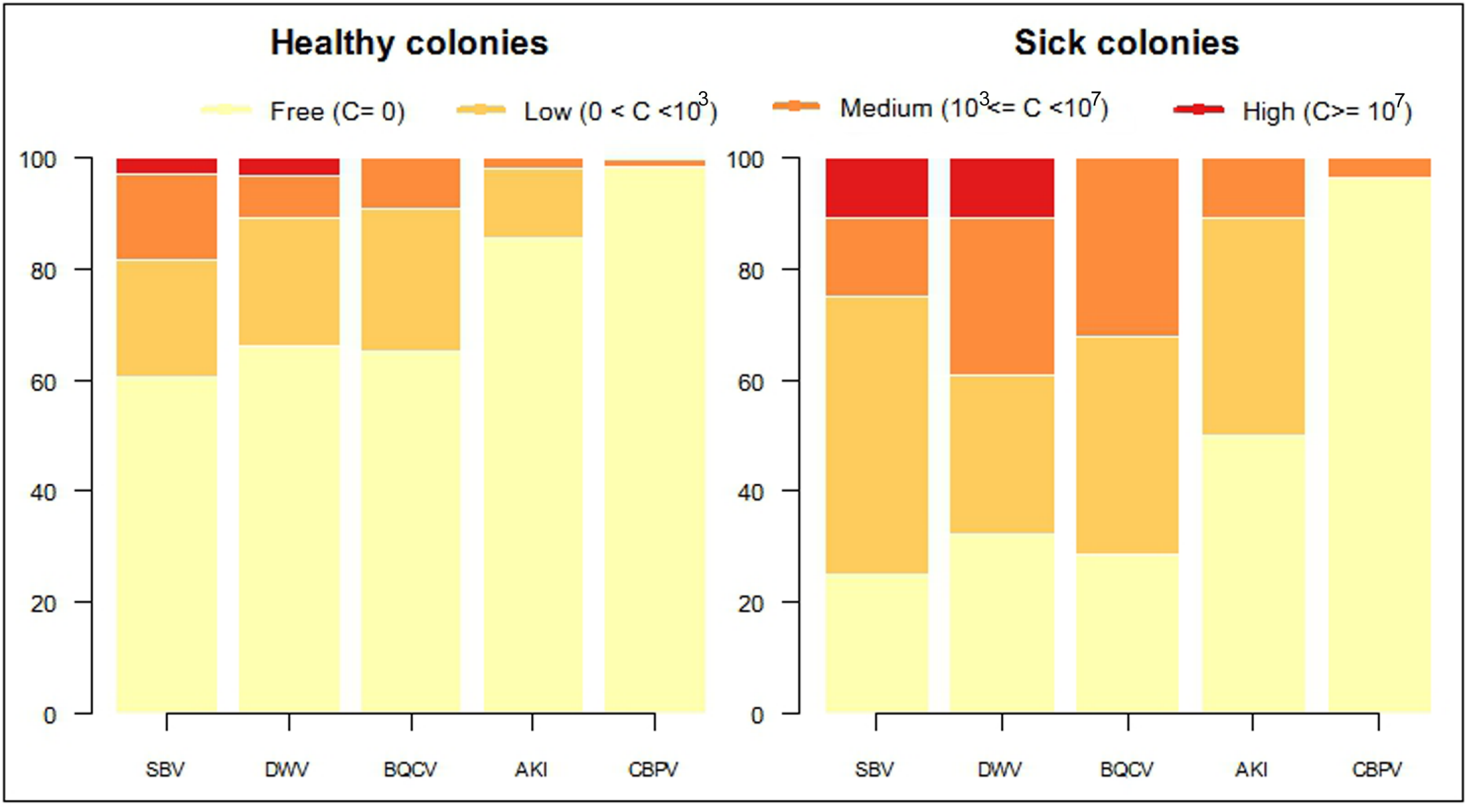
Percentage of viral titer categories for each virus in two groups of healthy and sick colonies.

For each investigated virus the samples of healthy and sick colonies were categorized according to the quantified virus titer. Beyond the two obvious categories, bees free of virus (copy number, C = 0) and symptomatic bees (C ≥ 10^7^), we decided to subdivide the remaining samples into two groups: samples with low virus titer (0 < C < 10^3^) and samples with medium virus titer (10^3^ ≤ C < 10^7^). For both DWV and SBV we found samples falling in all four categories, whereas high BQCV and AKI titers were never observed. Finally, CBPV was observed only in the two categories no virus or medium virus level. The distribution of samples within these four categories is displayed in Fig 4 for all viruses under investigation, demonstrating significant differences between healthy and sick colonies for the virus titers of SBV, DWV, BQCV and AKI (*P* < 0.001), but not for CBPV (*P* = 0.3842).

## Discussion

We report data on the prevalence of viruses in samples of healthy Danish honey bee colonies collected in the spring time. Similar to earlier studies from other countries [29, 30], no virus was found in 36% (n = 86) of the healthy colonies. In spite of us requesting bees to be collected from healthy colonies, a few samples contained at least one bee with a high virus titer. Instead of interpreting this as a beekeeper’s mistake, rather this result is expected since the virus is probably present at high levels in just a few bees in most colonies, however in samples of 20 bees it mostly goes undetected.

***Sacbrood virus*** is found to be the most prevalent virus in Danish honey bees for the period of investigation. Almost 40% of the healthy colonies carry SBV, with 18% of the population infected by a medium or high titer of the virus. This virus was even more frequently detected in our samples of sick colonies (75%) which is in accordance with a previous study from Denmark (81% [31]). Sacbrood is known as a disease affecting the brood of the honey bee, but it has also been reported from adult honey bees lacking any obvious sign of disease [14, 30, 34]. The occurrence of Sacbrood in the spring and summer may be related to colony growth with a high ratio of brood to nurse bees [55], or the quality of available pollen [29]. Even though an association between SBV and *Varroa* mites was reported in different studies [29, 56], the effect is most likely resulting from stress at colony level, since the disease occurs in larvae before these are attractive to *Varroa* mites. In Denmark it has frequently been reported that colonies suffer from severe outbreaks of SBV, not commonly found elsewhere in Europe [57].

***Deformed wing virus*** is the other highly prevalent virus in this study. It was detected in 34% of the healthy colonies with almost 11% of these carrying a medium or high titer which is associated with reduced life span of the adult bees [58]. This result is concurrent with previous reports from Denmark [13, 31, 49]. DWV has also been found to be highly prevalent in honey bees in other countries [12, 22, 30]. DWV can be detected in all developmental stages and castes of bees [29, 59]. It is closely associated with the *Varroa* mite and strongly suspected to be one of the biological agents for honeybee colony losses [12, 59], since a significant relationship between DWV and colony collapse was reported in several studies [10, 11, 13, 34, 50, 60]. Our data for the sick colonies (68% prevalence with 40% medium and high titer) may be associated with high levels of *Varroa* mites infestation during the preceding winter.

***Black queen cell virus*** is another highly prevalent virus in the healthy colonies in this study. It has been reported as a common virus in adult European honey bees [30], however, in Denmark only one single case was reported [31]. We hypothesize that the absence of BQCV positive sample in the previous study may be the result of lower sensitivity of the technique they used. We observed only low and medium titer infections, in both the healthy and the sick colonies.

BQCV has been detected with high titers in collapsed colonies [10]. A recent study suggests that BQCV has limited effects on both drone and worker health [61]. There are reports regarding a close association between BQCV and *N*. *apis* [25, 29]. As yet, no synergistic interaction has been found between BQCV and *N*. *ceranae* [61].

***Acute bee paralysis virus*, *Kashmir bee virus and Israeli bee paralysis virus*** are three closely related viruses that were analyzed together in a single assay [51]. Almost 14% of the healthy colonies were found positive, but only 2% of colonies carried a medium titer of virus and not a single case with high titer was detected. All three viruses are equally and highly virulent and have a close association with the *Varroa* mite [13, 20, 21, 28]. While our results do not distinguish between the three subtypes (ABPV, KBV and IAPV), all three are known to rapidly kill the bees. Several studies in recent years have identified the family of Acute paralysis viruses as one of the major biological agents detected in collapsed colonies [3, 13, 62]. Our results are in agreement with previous studies that report a low prevalence of these viruses in spring [13, 34], and observe high viral titers only in collapsing colonies [10, 30]. Therefore, high titers of these viruses are rarely detected in active survey studies since highly infected bees die fast and are therefore not sampled.

***Chronic bee paralysis virus*** is the rarest detected virus, also amongst the sick colonies. CBPV is known as a disease of adult honey bees, mostly with outbreaks during high nectar flow [32]. We only detected CBPV in 1.7% of the healthy colonies with a medium titer. This result is in agreement with previous studies that also reported a low prevalence of CBPV [29, 31].

Several of the viruses we discuss above are considered serious pathogens in regard to their impact on colony survival [10, 12, 13]. However, viruses often persist in colonies as covert infections [26] with limited consequences for colony health, and of little concern to beekeepers. Nonetheless, the standard procedure in virus analysis thus far has been to report the result as “negative” or “positive” [12, 59, 63]. In fact, a positive result from a whole-body extraction of 20 workers, as in our study, could result from a covert infection. Thus, a positive qualitative result does not necessarily allow for a meaningful prognosis.

For instance, even after successful *Varroa* treatment, DWV seems to prevail in the colony and the infection level will build up again, when the *Varroa* population increases. Individual bees parasitized by *Varroa* mites, either in the pupal stage or as adults, exhibit extraordinary high viral titers [64]. As the *Varroa* population in a colony increases, more and more bees carry such high infection levels and therefore the chance of including one of those bees in a random sample will also increase. Similar patterns are expected for the Acute bee paralysis virus complex, which may result in colony loss [65].

On the other hand, a virus like CBPV, which is not vectored by *Varroa*, may spread readily between colonies and is considered very contagious [32, 66]. We therefore consider it helpful if colonies carrying high virus titers within an apiary can be identified and adequately taken care of, for instance, by removing them to a quarantine apiary. In our experience beekeepers and breeders listen more readily and conceive the meaning of virus analysis results more easily when these are communicated as categories.

Frequently, due to the non-normal distribution of virus titers over several magnitudes, problems arise when trying to statistically compare two sets of samples. Categorization of quantitative virus titers renders the results accessible to statistical evaluation beyond non-parametric tests and thus allows comparisons between different samplings. Such data enable monitoring the progression of a viral infection in a colony or apiary, or comparisons between different breeding lines in a selection program. Based on a sufficient number of observations, critical thresholds will emerge that can be used for management decisions [57]. Especially in ongoing selection programmes for *Varroa* tolerance [42, 67] information on the virus load of potential queen and drone mother colonies may be provide useful information in the evaluation of breeding stock.
